# A population-based study of TyG index distribution and its relationship to cardiometabolic risk factors in children and adolescents

**DOI:** 10.1038/s41598-021-03138-6

**Published:** 2021-12-08

**Authors:** Jong Seo Yoon, Young Suk Shim, Hae Sang Lee, Il Tae Hwang, Jin Soon Hwang

**Affiliations:** 1 Department of Pediatrics, Hallym University Kangdong Sacred Heart Hospital, Hallym University College of Medicine, Seoul, Korea; 2 Department of Pediatrics, Ajou University School of Medicine, Ajou University Hospital, San 5, Wonchon-dong, Yeongtong-gu, Suwon, 443-721 Korea

**Keywords:** Endocrine system and metabolic diseases, Predictive markers, Paediatric research

## Abstract

The purpose of this study was to present age- and sex-specific distributions of the triglyceride-glucose (TyG) index and to evaluate their relationship with cardiometabolic risk factors in children and adolescents. A total of 7404 participants aged 10–18 years from the Korean National Health and Nutrition Survey were included as the reference population. The TyG index was calculated as ln(fasting triglyceride [mg/dL] × fasting glucose [mg/dL]/2). The percentile of the TyG index exhibited a steady linear relationship with age for both sexes. TyG index significantly correlated with waist circumference (WC) standard deviation score (SDS; r = 0.110, *p* < 0.001), systolic blood pressure (SBP; r = 0.104, *p* < 0.001), diastolic blood pressure (DBP; r = 0.083, *p* < 0.001), glucose (r = 0.220, *p* < 0.001), high-density lipoprotein cholesterol (HDL-C; r = − 0.325, *p* < 0.001), and triglycerides (TG; r = 0.926, *p* < 0.001). Multiple linear regression analysis revealed that the TyG index was significantly associated with WC SDS (β = 0.116, *p* < 0.001), SBP (β = 2.009, *p* < 0.001), DBP (β = 1.464, *p* < 0.001), glucose (β = 3.376, *p* < 0.001), HDL-C (β =  − 6.431, *p* < 0.001), and TG (β = 85.518, *p* < 0.001). Our results suggest that the TyG index has a steady linear distribution for sex and age in children and adolescents and constitutes an indicator for predicting metabolic disorders that could lead to cardiovascular disease later in life.

## Introduction

Cardiovascular disease (CVD), a leading cause of morbidity and mortality, is a global disease burden^1^. Hyperglycemia, central obesity, elevated triglycerides, decreased high-density lipoprotein (HDL), and elevated blood pressure (BP) are components of metabolic syndrome (MetS), and each factor may independently increase the risk of CVD^2,3^. These risk factors can even cluster in children and increase the risk of CVD, eventually leading to the onset of CVD in youth^4,5^. Therefore, the early identification of high-risk groups and lifestyle modifications are necessary to prevent disease progression. Insulin resistance (IR) plays a major role in the pathogenesis of CVD^6^. The homeostatic model assessment for insulin resistance (HOMA-IR), a commonly used method for evaluating IR, is a clinical predictor of the development of MetS, type 2 diabetes mellitus (T2DM), and CVD in youth and adults^7–11^. However, it is difficult to assess IR in children and adolescents because of physiological characteristics that temporarily increase IR during puberty^12,13^. HOMA-IR in pediatric populations is not evenly distributed by sex, age, and BMI standard deviation score (SDS), and there is no consensus on the normal reference range. Therefore, there is currently no consensus for diagnosing diseases related to IR and evaluating disease status.

The TyG index has emerged as a reliable surrogate marker for IR. The TyG index is significantly associated with the presence of T2DM, nonalcoholic fatty liver disease (NAFLD), and MetS in adults and is a better predictor of IR than HOMA-IR^14–18^. Recent studies have reported that the TyG index can predict the presence and progression of coronary artery calcification (CAC) in adults and is associated with cardiovascular risk factors in children and adolescents^19–22^. Cutoff points for the TyG index for MetS and cardiometabolic risk have been suggested in many studies of children and adolescents, and the usefulness of screening has been sufficiently emphasized^22–25^. For the TyG index to be useful in clinical practice, it is necessary to determine its association with various confounding factors and whether it is consistently distributed in children and adolescents; however, studies on these characteristics are still lacking. A detailed distribution of the TyG index in children and adolescents has not yet been presented, and the effects of clinical confounders, such as age, sex, and BMI SDS, are unknown.

Therefore, this study aimed to present a detailed distribution and curve for the TyG index in children and adolescents and investigate whether confounding factors affect its ability to evaluate cardiometabolic risk factors.

## Results

### Clinical characteristics of the study population

The clinical characteristics of the reference population (3,945 boys and 3,459 girls) are presented as mean values ± standard deviation or frequency (%) (Table 1). The mean ages of the boys and girls were 13.77 ± 2.50 and 13.85 ± 2.51 years, respectively. Height SDS, weight SDS, SBP, DBP, and serum biochemistry results were lower in boys than in girls (*P* < 0.01). The mean TyG index was higher in boys (8.09 ± 0.52) than in girls (8.15 ± 0.48) (*P* < 0.001). Boys were more likely to be alcohol drinkers and smokers and engage in a higher frequency of physical activity than girls (all *P* < 0.01).

**Table 1:** Clinical characteristics of study population (*n* = 7404).

	Boys (*n* = 3945)	Girls (*n* = 3459)	*P*
Age, y	13.77 ± 2.50	13.85 ± 2.51	0.169
Height, cm	163.44 ± 12.65	156.77 ± 8.28	< 0.001
Height, SDS	0.80 ± 1.06	0.53 ± 1.04	< 0.001
Weight, kg	56.70 ± 15.34	50.06 ± 11.06	< 0.001
Weight SDS	0.43 ± 1.04	0.30 ± 1.05	< 0.001
BMI, kg/m^2^	20.91 ± 3.84	20.19 ± 3.38	< 0.001
BMI SDS	0.11 ± 1.08	0.10 ± 1.06	0.681
WC, cm	71.44 ± 10.58	67.08 ± 8.40	< 0.001
WC SDS	− 0.17 ± 1.13	− 0.14 ± 1.0	0.189
SBP, mmHg	108.69 ± 10.65	104.10 ± 9.28	< 0.001
DBP, mmHg	66.49 ± 9.55	65.50 ± 8.30	< 0.001
Glucose, mg/dL	90.54 ± 7.16	89.24 ± 8.15	< 0.001
T-C, mg/dL	155.30 ± 26.97	163.17 ± 26.15	< 0.001
HDL-C, mg/dL	49.80 ± 9.89	52.11 ± 9.86	< 0.001
TG, mg/dL	82.93 ± 47.34	86.49 ± 44.09	0.001
LDL-C, mg/dL	88.92 ± 23.21	93.76 ± 22.80	< 0.001
TyG index	8.09 ± 0.52	8.15 ± 0.48	< 0.001
Alcohol drinker (%)	219 (5.55)	135 (3.90)	0.001
Smoker (%)	621 (15.74)	234 (6.76)	< 0.001
Household income ≤ 1st quartile (%)	435 (11.03)	384 (11.10)	0.948
Rural residence (%)	655 (16.60)	577 (16.68)	0.953
Physically active (%)	2094 (53.08)	1510 (43.65)	< 0.001

SDS, standard deviation score; WC, waist circumference; BMI, body mass index; SBP, systolic blood pressure; DBP, diastolic blood pressure; T-C, total cholesterol; HDL-C, high-density lipoprotein cholesterol; LDL-C, low-density lipoprotein cholesterol; TyG index, triglyceride–glucose index.

### Reference interval of the TyG index in the study population using the LMS method

**Figure 1:**
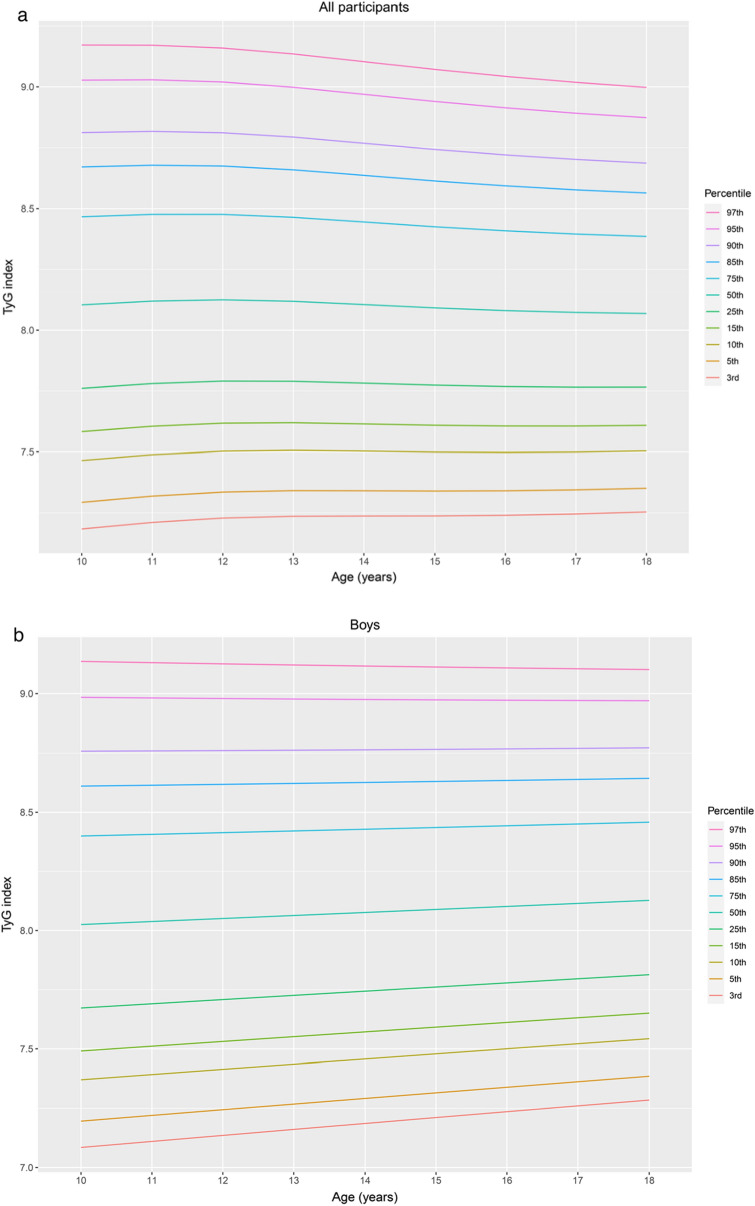

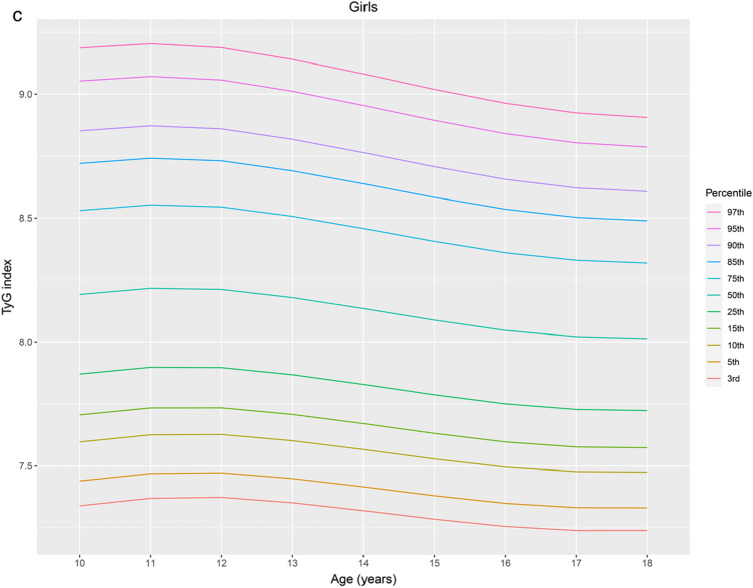
Percentile curves for the TyG index for all participants (**a**), boys (**b**) and girls (**c**) aged 10–18 years. The reference intervals for the age-specific TyG index are presented as the following percentile values: 3rd, 5th, 10th, 15th, 25th, 50th, 75th, 85th, 90th, 95th, and 97th, as indicated in different colors. TyG index, triglyceride–glucose index.

Percentiles and L, M, and S values of the TyG index by sex and age for the study population aged 10–18 years are presented in Table 2. The TyG index distribution charts for all participants, boys, and girls were stably distributed (Fig. 1a, b, and c, respectively). The median TyG index values of the boys and girls remained nearly constant at approximately 8.07 and 8.14, respectively. In girls, the TyG index exhibited a steady linear distribution across all ages and a steady linear decrease from 11 to 18 years. In boys, the TyG index also presented a steady linear distribution at all ages and a steady increase at an age below the 50th percentile. For all participants, the median TyG index remained nearly constant at approximately 8.11. The TyG index exhibited a steady linear distribution across all ages and a slight decline from 11 to 18 years over the 50th percentile.

**Table 2:** Reference interval of the triglyceride–glucose (TyG) index in the study population according to the LMS method.

Age	*n*	L	M	S	Percentile										
					3rd	5th	10th	15th	25th	50th	75th	85th	90th	95th	97th
**All participants**															
10	814	− 0.210	8.104	0.064	7.184	7.293	7.465	7.583	7.761	8.104	8.467	8.670	8.811	9.027	9.172
11	895	− 0.210	8.119	0.063	7.210	7.319	7.488	7.605	7.780	8.119	8.477	8.677	8.816	9.029	9.171
12	882	− 0.210	8.125	0.062	7.228	7.335	7.502	7.617	7.791	8.125	8.477	8.674	8.811	9.020	9.159
13	913	− 0.210	8.119	0.061	7.236	7.341	7.506	7.619	7.790	8.119	8.465	8.658	8.793	8.998	9.135
14	929	− 0.210	8.105	0.060	7.237	7.341	7.503	7.614	7.782	8.105	8.445	8.635	8.768	8.969	9.103
15	800	− 0.210	8.092	0.060	7.237	7.339	7.499	7.609	7.774	8.092	8.426	8.612	8.742	8.940	9.071
16	752	− 0.210	8.081	0.059	7.240	7.340	7.498	7.606	7.768	8.081	8.409	8.592	8.720	8.913	9.043
17	762	− 0.210	8.073	0.058	7.245	7.344	7.499	7.606	7.765	8.073	8.396	8.576	8.701	8.891	9.018
18	657	− 0.210	8.069	0.057	7.253	7.351	7.504	7.608	7.766	8.069	8.386	8.563	8.686	8.873	8.998
**Boys**															
10	439	− 0.306	8.025	0.067	7.085	7.196	7.371	7.491	7.673	8.025	8.400	8.611	8.758	8.984	9.136
11	482	− 0.306	8.038	0.066	7.110	7.220	7.392	7.511	7.690	8.038	8.407	8.614	8.759	8.982	9.131
12	483	− 0.306	8.051	0.065	7.136	7.244	7.414	7.531	7.708	8.051	8.414	8.618	8.761	8.979	9.126
13	493	− 0.306	8.064	0.064	7.161	7.268	7.436	7.552	7.726	8.064	8.421	8.622	8.762	8.977	9.121
14	492	− 0.306	8.076	0.063	7.186	7.292	7.458	7.572	7.744	8.076	8.428	8.626	8.764	8.975	9.116
15	438	− 0.306	8.089	0.062	7.211	7.315	7.479	7.592	7.761	8.089	8.436	8.630	8.766	8.973	9.112
16	380	− 0.306	8.102	0.061	7.236	7.339	7.500	7.611	7.779	8.102	8.443	8.635	8.768	8.972	9.108
17	383	− 0.306	8.115	0.060	7.261	7.362	7.522	7.631	7.796	8.115	8.450	8.639	8.770	8.971	9.105
18	355	− 0.306	8.127	0.059	7.285	7.386	7.543	7.651	7.813	8.127	8.458	8.644	8.773	8.970	9.102
**Girls**															
10	375	− 0.381	8.191	0.060	7.339	7.439	7.597	7.706	7.871	8.191	8.530	8.720	8.852	9.053	9.186
11	413	− 0.381	8.216	0.059	7.369	7.469	7.626	7.734	7.898	8.216	8.553	8.741	8.872	9.071	9.204
12	399	− 0.381	8.212	0.058	7.373	7.472	7.627	7.734	7.896	8.212	8.545	8.731	8.860	9.057	9.188
13	375	− 0.381	8.179	0.058	7.351	7.449	7.602	7.708	7.868	8.179	8.507	8.690	8.818	9.012	9.141
14	437	− 0.381	8.135	0.057	7.319	7.415	7.566	7.671	7.829	8.135	8.458	8.638	8.764	8.954	9.081
15	362	− 0.381	8.088	0.057	7.285	7.380	7.529	7.631	7.787	8.088	8.406	8.584	8.707	8.894	9.019
16	372	− 0.381	8.047	0.056	7.256	7.349	7.496	7.597	7.750	8.047	8.360	8.535	8.656	8.841	8.963
17	379	− 0.381	8.021	0.056	7.239	7.331	7.477	7.577	7.728	8.021	8.330	8.502	8.622	8.804	8.924
18	302	− 0.381	8.013	0.055	7.240	7.331	7.474	7.573	7.723	8.013	8.319	8.489	8.607	8.787	8.906

### Unadjusted and adjusted correlations between the TyG index and clinical variables

Correlations between the TyG index and clinical variables, including sex; age; BMI SDS; WC SDS; SBP; DBP; glucose, T-C, HDL-C, TG, LDL-C levels; alcohol drinking; smoking; household income; rural residence; and physical activity, are presented in Table 3. In the adjusted Pearson’s correlation analyses, after controlling for sex, age, and BMI SDS in all participants (adjusted model 2), the TyG index was positively correlated with WC SDS (r = 0.110,* P* < 0.001); SBP (r = 0.104, *P* < 0.001); DBP (r = 0.083, *P* < 0.001); and glucose (r = 0.220, *P* < 0.001), T-C (r = 0.241, *P* < 0.001), TG (r = 0.926, *P* < 0.001), and LDL-C (r = 0.051, *P* < 0.001) levels, but negatively correlated with HDL-C levels (r =  − 0.325, *P* < 0.001). After controlling for age and BMI SDS, the correlations between the TyG index and the above variables in boys and girls were similar to the above results (adjusted model 4).

**Table 3:** Unadjusted and adjusted correlations between the triglyceride–glucose (TyG) index and cardiometabolic risk factors in the study population (*n* = 7,404).

	Coefficient (r)								
	All participants			Boys			Girls		
	Unadjusted	Adjusted		Unadjusted	Adjusted		Unadjusted	Adjusted	
		Model 1	Model 2		Model 3	Model 4		Model 3	Model 4
Sex	0.051*	–	–	–	–	–	–	–	–
Age, y	–0.035**	–	–	0.060*	–	–	− 0.154*	–	–
BMI SDS	0.246**	0.245*	–	0.276*	0.282*	–	0.209*	0.207*	–
WC SDS	0.267*	0.266*	0.110*	0.301*	0.303*	0.117*	0.222*	0.228*	0.100*
SBP, mmHg	0.141*	0.169*	0.104*	0.187*	0.178*	0.089*	0.113*	0.128*	0.082*
DBP, mmHg	0.086*	0.106*	0.083*	0.133*	0.119*	0.089*	0.027	0.067*	0.049
Glucose, mg/dL	0.240*	0.244*	0.220*	0.210*	0.224*	0.192*	0.287*	0.267*	0.251*
T-C, mg/dL	0.273*	0.266*	0.241*	0.292*	0.308*	0.268*	0.239*	0.238*	0.227*
HDL-C, mg/dL	− 0.350*	− 0.365*	− 0.325*	− 0.340*	− 0.336*	− 0.286*	− 0.383*	− 0.382*	− 0.350*
TG, mg/dL	0.931*	0.931*	0.926*	0.928*	0.928*	0.922*	0.934*	0.933*	0.930*
LDL-C, mg/dL	0.098*	0.091*	0.051*	0.105*	0.115*	0.055*	0.078*	0.079*	0.057*

BMI, body mass index; SDS, standard deviation score; WC, waist circumference; SBP, systolic blood pressure; DBP, diastolic blood pressure; T–C, total cholesterol; HDL-C, high-density lipoprotein cholesterol; TG, triglycerides; LDL, low-density lipoprotein cholesterol.

Adjusted model 1: adjusted for sex and age.

Adjusted model 2: adjusted for sex, age, and BMI SDS.

Adjusted model 3: adjusted for age.

Adjusted model 4: adjusted for age and BMI SDS.

* *P* < 0.001, ** *P* < 0.01.

### Association between the TyG index and cardiometabolic risk factors on multiple linear regression analysis

Results of the multiple linear regression analysis of the TyG index and WC SDS; SBP; DBP; and glucose, T-C, HDL-C, TG, and LDL-C levels are shown in Table 4. After controlling for sex, age, BMI SDS, alcohol drinking, smoking, household income, rural residence, and physical activity among all participants, WC (β = 0.116, *P* < 0.001); SBP (β = 2.009, *P* < 0.001); DBP (β = 1.464, *P* < 0.001); and glucose (β = 3.376, *P* < 0.001), T-C (β = 13.012, *P* < 0.001), HDL-C (β =  − 6.431, *P* < 0.001), TG (β = 85.518, *P* < 0.001), and LDL-C (β = 2.339, *P* < 0.001) levels were significantly associated with the TyG index. In boys and girls, multiple linear regression analysis after controlling for age, BMI SDS, alcohol drinking, smoking, household income, rural residence, and physical activity showed that these cardiometabolic risk factors were also significantly associated with the TyG index, similar to the results for the whole population.

**Table 4:** Association between the triglyceride–glucose (TyG) index and cardiometabolic risk factors using multiple linear regression analysis in the study population (*n* = 7,404).

	β	SE	R^2^	*P*
	All participants^1^			
WC SDS	0.116	0.012	0.788	< 0.001
SBP, mmHg	2.009	0.224	0.187	< 0.001
DBP, mmHg	1.464	0.203	0.123	< 0.001
Glucose, mg/dL	3.376	0.175	0.105	< 0.001
T-C, mg/dL	13.012	0.611	0.109	< 0.001
HDL-C, mg/dL	− 6.431	0.217	0.179	< 0.001
TG, mg/dL	85.518	0.405	0.866	< 0.001
LDL-C, mg/dL	2.339	0.544	0.049	< 0.001
**Boys**^2^				
WC SDS	0.108	0.015	0.838	< 0.001
SBP, mmHg	1.681	0.302	0.226	< 0.001
DBP, mmHg	1.570	0.281	0.166	< 0.001
Glucose, mg/dL	2.679	0.220	0.092	< 0.001
T-C, mg/dL	13.994	0.805	0.142	< 0.001
HDL-C, mg/dL	− 5.396	0.287	0.188	< 0.001
TG, mg/dL	84.767	0.569	0.861	< 0.001
LDL-C, mg/dL	2.437	0.720	0.073	< 0.001
**Girls**^3^				
WC SDS	0.123	0.021	0.730	< 0.001
SBP, mmHg	1.595	0.330	0.074	< 0.001
DBP, mmHg	0.869	0.296	0.070	0.003
Glucose, mg/dL	4.309	0.285	0.108	< 0.001
T-C, mg/dL	12.873	0.939	0.057	< 0.001
HDL-C, mg/dL	− 7.261	0.332	0.173	< 0.001
TG, mg/dL	86.674	0.582	0.873	< 0.001
LDL-C, mg/dL	2.800	0.836	0.016	< 0.001

WC, waist circumference; SDS, standard deviation score; SBP, systolic blood pressure; DBP, diastolic blood pressure; T-C, total cholesterol; HDL-C, high-density lipoprotein cholesterol; LDL, low-density lipoprotein cholesterol; triglyceride–glucose index, TyG index.

^1^Multiple linear regression analysis of the TyG index and cardiometabolic risk factors was performed after controlling for sex, age, body mass index (BMI) SDS, alcohol drinking, smoking, household income, rural residence, and physical activity among all participants.

^2^Multiple linear regression analysis of the TyG index and cardiometabolic risk factors was performed after controlling for age, BMI SDS, alcohol drinking, smoking, household income, rural residence, and physical activity among all boys.

^3^Multiple linear regression analysis of the TyG index and cardiometabolic risk factors was performed after controlling for sex, age, BMI SDS, alcohol drinking, smoking, household income, rural residence, and physical activity among all girls.

## Discussion

The main finding of the present study was that the TyG index showed a steady linear distribution in both boys and girls and differed significantly by sex. The TyG index was independently significantly associated with WC SDS; SBP; DBP; and glucose, T-C, TG, LDL-C, and HDL-C levels after adjustment for sex, age, and BMI SDS.

With the increase in childhood obesity worldwide, so does the incidence of MetS and T2DM^26–28^. These problems persist until adulthood, leading to complications such as T2DM and CVD; therefore, it is necessary to prevent disease progression using early detection. Obesity is considered a risk factor for IR, a common component of the shared pathology of MetS, T2DM, and CVD; therefore, it is necessary to measure IR early in overweight and obese children^29,30^. The hyperinsulinemic euglycemic clamp is the gold standard method of insulin resistance measurement; however, it is time-consuming and expensive, limiting its clinical use^31^. Fasting plasma insulin, HOMA-IR, and QUICKI are the most commonly used insulin-resistant surrogate markers^7^. These tests are currently useful for evaluating IR; however, they have the following limitations in youth: (1) a reliable reference range has not yet been proposed because it is difficult to determine normal values and cutoff points due to a lack of longitudinal evidence in youth for predicting cardiovascular outcome risk and (2) the values vary according to pubertal development and ethnic differences^8^. In addition, universal insulin assay standardization is lacking because of variable insulin measurement techniques among laboratories^32^.

The TyG index has recently emerged as a surrogate marker for IR^15,23,33–35^. In adults, it is useful for predicting T2DM, MetS, NAFLD, and CVD and is superior to the HOMA-IR index. Many studies have evaluated the relationship between the TyG index and CVD. The TyG index is independently associated with the presence and progression of CAC, which is an effective marker for atherosclerosis-related CVD^36^. Although the mechanism underlying the association between the TyG index level and CVD risk factors remains unclear, IR is a major contributor to CVD, and the TyG index is a reliable surrogate marker for IR, which may indirectly explain this relationship. An elevated TyG index is significantly related to elevated BP in healthy children aged 6–15 years^37^. In Brazilian children aged 4–9 years, overweight, total body fat and central fat, increased BP, and worsening lipid profile were associated with an increased TyG index. Although atherosclerotic CV events rarely occur in youth, atherogenic processes that lead to CVD begin in childhood and progress throughout life^5^. Therefore, establishing healthy behaviors for the heart and managing CV risk factors in childhood may prevent or delay the development of atherosclerosis and, in turn, reduce the risk of CVD later in life.

One of the reasons why it is difficult to assess cardiometabolic risk factors in children and adolescents is that the criteria are varied and differ slightly, as suggested by Cook, de Ferranti, and the International Diabetes Federation^38^. In the present study, cardiometabolic risk factors, such as central obesity, hypertension, dyslipidemia, and hyperglycemia, known independent risk factors for CVD, were independently associated with the TyG index after controlling for confounding factors such as age, sex, and BMI. Therefore, using only fasting blood glucose and TG levels, the TyG index enables a comprehensive and simple assessment of several cardiovascular risk factors in children and adolescents.

Although the TyG index is certainly a reliable and useful test for detecting IR-related diseases such as CVD, it is necessary to confirm its limitations in childhood and adolescence, such as with the HOMA-IR, before applying it to clinical practice. In this study, detailed distributions and smooth curves using the LMS method were presented for boys, girls, and all participants aged 10–18 years. The TyG index showed a stable distribution that differed from the previously reported distribution for HOMA-IR. The conventional IR evaluation method using insulin could not provide a diagnostic value for pediatric IR and a value that can represent disease progression because IR fluctuates greatly with confounding factors such as age, sex, and BMI. The difference in the distribution of surrogate markers using insulin is that the TyG index in the present study showed a stable distribution, even though there was a distribution difference between boys and girls. In future studies, it will be necessary to explore the effect of the difference in distribution between boys and girls in determining the cutoff value for predicting IR-related diseases. If the difference in distribution between the sexes does not affect the TyG index cutoff value, the previously reported TyG cutoff values will provide reliable values for clinical application and will be useful for assessing disease progression in children and adolescents in the future.

The results of the present study are valuable as a large nationally representative study population, but there are some limitations. First, because the data obtained from the KNHANES does not contain information about Tanner stage or sex hormones levels and does not provide data for children under 10 years of age, the differences in TyG index between pubertal and prepubertal periods cannot be compared. The comparison of TyG index distributions between the prepubertal and pubertal periods is thus required in further research. Despite these limitations, our results are meaningful as a practical reference for tracking children and adolescents aged > 10 years, the age at which pediatricians evaluate IR-related diseases. Second, because we analyzed the cross-sectional data of only Korean children and adolescents, our results cannot be directly applicable to other races. Therefore, we propose further research in other less homogeneous countries to determine the characteristics of the curves and distributions of the TyG index in different ethnic groups.

Although the TyG index is recognized as a good surrogate marker for indirectly measuring IR and is thought to be useful and convenient in children and adolescents, its characteristics have not been studied with respect to various clinical variables. To the best of our knowledge, this study is the first to analyze the TyG index characteristics related to clinical variables and to evaluate the characteristics of the TyG index distribution in children and adolescents. Here, we evaluated the value of the TyG index as a surrogate insulin resistant marker that can overcome the limitations of IR assessment caused by differences in the physical characteristics with age in children and adolescents.

In conclusion, this pediatric population-based study confirmed that the TyG index has clinical value by showing a stable distribution in children and adolescents regardless of age, sex, and BMI. In addition, this study proved that the TyG index is a surrogate marker for insulin resistance because it can individually or comprehensively evaluate various cardiovascular risk factors based only on fasting blood sugar and TG levels without the complexity of evaluating risk factors by considering sex, age, and BMI SDS.

## Methods

### Study population

Statistical analyses were performed using data obtained from the Korea National Health and Nutrition Examination Survey (KNHANES) 2008–2016; a total of 81,503 subjects were registered. The KNHANES, a population-based cross-sectional survey, was designed to assess the health and nutritional status of a nationally representative non-institutionalized Korean sample^37^. This survey used a stratified multilevel probability sampling method to select participants by household units. The Korean national survey consists of three parts: a health questionnaire, a health checkup, and a nutritional assessment. We included 9287 children and adolescents aged 10–18 years in the preliminary analyses. Participants for whom anthropometric variables or health questionnaire responses (n = 685) or BMI and BP data (n = 1186) were unavailable were excluded. We also excluded participants with triglyceride (TG) levels ≥ 400 mg/dL (n = 13) because the low-density lipoprotein cholesterol (LDL-C) level was determined using Friedewald’s equation. Finally, 7417 participants aged 10–18 years were included in this study. All data sets from the KNHANES are publicly accessible at http://knhanes.cdc.go.kr. The study protocol for the KNHANES 2008–2016 was approved by the Institutional Review Board of the Korean Centers for Disease Control and Prevention. Informed consent was obtained from all KNHANES participants and their legal guardians. The KNHANES was performed in accordance with relevant guidelines and regulations. The present study was approved by the Institutional Review Board of Hallym University Kangdong Sacred Heart Hospital (IRB no. KANGDONG 2021-01-006).

### Measurements

Height, weight, WC, SBP, and DBP were determined according to standardized protocols. BMI was calculated as the ratio of weight to height squared (kg/m^2^). The SDS values for height, weight, WC, and BMI were assessed using the LMS (L, lambda for the Box–Cox power for skewness; M, mu for the median; S, sigma for the generalized coefficient of variation) method (SDS = [measured value/M]^1/L^/LS) based on the 2017 Korean national reference^40^. SBP and DBP (mmHg) were evaluated in the right upper arm three times using a calibrated sphygmomanometer (Baumanometer Desk model 0320, Baum, NY, USA) and an appropriately sized cuff. Measurements were conducted at 2-min intervals. The mean of the last two values was used as SBP and DBP in the analyses.

Blood samples were obtained from the antecubital vein after the participants had fasted for at least 8 h. The collected blood samples were analyzed within 24 h in a central laboratory (NeoDin Medical Institute, Seoul, Korea). Biochemical tests, including glucose, total cholesterol (T-C), HDL cholesterol (HDL-C), and TG levels, were performed using an automatic analyzer (Hitachi 7600, Hitachi, Tokyo, Japan). The LDL-C level (mg/dL) was calculated using Friedewald’s equation^41^. The TyG index was calculated as follows: ln(fasting TG [mg/dL] × fasting glucose [mg/dL]/2)^33^.

### Collection of socioeconomic characteristics and medical history

Lifestyle-related variables included alcohol consumption, smoking, physical activity, household income, and residence. Alcohol drinkers were defined as boys and girls who consumed two or more alcoholic beverages per month in the previous year, and participants were divided into two groups (drinkers versus non-drinkers). Smokers were defined as those who smoked five or more packs of cigarettes during their lifetime, and participants were divided into two groups (smokers versus nonsmokers). Physical activity was defined as one or more of the following: (1) vigorous physical activity for at least 3 days for 20 min per week; (2) moderate physical activity, at least 5 days for 30 min per week; and (3) light physical activity, walking for at least 5 days for 30 min per week. Subjects answered yes or no for each of the three types of physical activity described above. Upon answering yes to any of them, a subject was classified into the physical activity group. Household income is presented in quartiles, and the subjects were divided into two groups (lowest quartile or second lowest quartile and above). Participants’ areas of residence were divided into two groups (urban versus rural).

### Statistical analysis

R version 3.6.1 for Windows (The R Foundation for Statistical Computing, Vienna, Austria) was used to perform the statistical analyses. The parameters of LMS for the TyG index were determined using the GAMLSS package^42^. The reference interval for the sex- and age-specific TyG index was obtained using the LMS method and is presented as the following percentile values: 3rd, 5th, 10th, 15th, 25th, 50th, 75th, 85th, 90th, 95th, and 97th percentiles. To determine statistical significance, analysis of variance and chi-squared tests were used to examine continuous and categorical variables according to sex- and age-specific TyG index groups. Continuous variables and categorical variables were presented as mean (standard deviation) and percentages (%). Unadjusted and adjusted Pearson’s correlation analyses were performed to investigate the correlation between the TyG index and clinical variables. In all participants, adjusted models included controls for sex and age (model 1) and for sex, age, and BMI SDS (model 2). In boys and girls, adjusted models 3 and 4 included controls for age, age, and BMI SDS, respectively. To evaluate the association between the TyG index and cardiometabolic risk factors including WC SDS, SBP, DBP, glucose, T-C, HDL-C, TG, and LDL-C levels, multiple linear regression analyses were conducted after the adjustment for sex, age, BMI SDS, alcohol drinking, smoking, household income, rural residence, and physical activity among all participants. In boys and girls, multiple linear regression analysis of the TyG index and cardiometabolic risk factors was performed after the adjustment for age, BMI SDS, alcohol drinking, smoking, household income, rural residence, and physical activity.
